# Assessing Disease Activity in Pediatric Chronic Nonbacterial Osteomyelitis: A Proposal for Composite Scoring, Including Inactivity Measures

**DOI:** 10.1002/acr2.70061

**Published:** 2025-07-02

**Authors:** Christiane Reiser, Jens Klotsche, Anja Schnabel, Christine Hofmann, Nadine Groesch, Martina Niewerth, Kirsten Minden, Hermann Girschick

**Affiliations:** ^1^ Division of Pediatric Rheumatology, Department of Pediatrics and Autoinflammation Reference Centre Tübingen, University Hospital Tübingen, Germany, and Department of Pediatrics, Landeskrankenhaus Bregenz Bregenz Austria; ^2^ Deutsches Rheuma‐Forschungszentrum Berlin, ein Institut der Leibniz‐Gemeinschaft and Charité – Universitätsmedizin Berlin, Freie Universität Berlin, Humboldt Universität zu Berlin, and Berlin Institute of Health Berlin Germany; ^3^ Department of Pediatrics, Faculty of Medicine and University Hospital Carl Gustav Carus, Technische Universität Dresden Dresden Germany; ^4^ Department of Pediatrics Landeskrankenhaus Bregenz Bregenz Austria; ^5^ Deutsches Rheuma‐Forschungszentrum Berlin, ein Institut der Leibniz‐Gemeinschaft Berlin Germany; ^6^ Deutsches Rheuma‐Forschungszentrum Berlin, ein Institut der Leibniz‐Gemeinschaft and Charité – Universitätsmedizin Berlin, Freie Universität Berlin, Humboldt Universität zu Berlin, and Berlin Institute of Health and German Center for Child and Adolescent Health Berlin Germany; ^7^ Vivantes Klinikum Friedrichshain, Children's Hospital, Berlin and Children's Hospital, University of Wuerzburg Wuerzburg Germany

## Abstract

**Objective:**

Chronic nonbacterial osteomyelitis (CNO) is an autoinflammatory bone disease with no validated criteria for assessing disease activity (DA), inactive disease, or remission. To date, DA assessment has relied on subjective judgments from patients, rheumatologists, and/or radiologists. Evaluations based on composite DA measures are emerging. The Pediatric CNO (PedCNO) response score documents relative DA changes during follow‐up in analogy to the Pediatric American College of Rheumatology score for juvenile idiopathic arthritis. The international Childhood Arthritis and Rheumatology Research Alliance (CARRA) CNO initiative proposed a numeric composite DA score (CDAS) on the basis of the patient global assessment of DA (PAG), patient assessment of pain (PAP), and clinically active CNO lesions. We aim to propose different composite scores for assessment of DA, including physician assessment of DA and magnetic resonance imaging (MRI) findings; to evaluate the previously published CNO CDAS and the PedCNO response score using established registry data; and to suggest a PedCNO 90% improvement (PedCNO90) category.

**Methods:**

Newly diagnosed patients with CNO were enrolled between 2015 and 2020 and analyzed for clinical course and DA measures (physician global assessment of DA [PGDA]/PAG and pain scores) for up to 4 years of follow‐up (YFU) in the National Pediatric Rheumatologic Database.

**Results:**

A total of 400 patients were enrolled. In single numeric scores only, clinical and MRI lesion scores reached significant changes (from baseline to 3 YFU: *P* = 0.003/*P* = 0.004). Composite scores, which include MRI DA scores consisting of one clinical patient‐based assessment parameter (PAG/PAP of DA), the PGDA and MRI lesion count are less dependent on subjective measures and demonstrate more pronounced changes over time of supposed CNO DA compared with the CNO CDAS. A PedCNO90 response score gradually increased during follow‐up, ultimately reaching a PedCNO90 in half of the remaining patients after 4 years.

**Conclusion:**

Composite scores, including MRI lesions and PGDA, seem to be promising tools for describing the activity of CNO and are proposed. The composition of DA scores seems essential for future studies.

## INTRODUCTION

In the treatment of chronic nonbacterial osteomyelitis (CNO), internationally consented measures of disease activity (DA) are lacking. Physicians face several unresolved challenges regarding disease management, including whether clinical monitoring and treatment decisions should be guided by magnetic resonance imaging (MRI), patient or parental reports of pain and global DA, or physicians’ assessment of DA.

The established Pediatric CNO (PedCNO) response score (consisting of the five variables erythrocyte sedimentation rate [ESR], number of radiologic lesions [here: MRI defined], physician global assessment of DA [PGDA; via the Numeric Rating Scale (NRS)], patient global assessment of DA [PAG; NRS] and the Childhood Health Assessment Questionnaire [C‐HAQ]) encompasses all these aspects, combining clinical and radiologic components.^1^ However, its use in daily clinical practice has been considered limited^2^: the complexity of calculation (PedCNO30, PedCNO50, or PedCNO70 score at least 30% 50%, or 70% improvement, respectively, in at least three of five core set variables, with no more than one of the remaining variables deteriorating by more than 30%, 50%, or 70%, respectively)^1^ may pose a challenge because the possibility of a single score parameter worsening during follow‐up without affecting the score calculation could limit its validity and relevance. The main limitation of the PedCNO score in the context of DA (analogous to the Pediatric American College of Rheumatology response^3^, ^4^) is that it reports only relative changes in DA, even though the score bases are absolute measurements. Moreover, the availability of repetitive whole‐body MRI (WB‐MRI) analyses, as requested, is not guaranteed in all centers. As a consequence, an alternative scoring system has been proposed: the Childhood Arthritis and Rheumatology Research Alliance (CARRA) recently introduced the CNO composite DA score (CDAS), which focuses on patient and parental considerations.^2^ MRI‐based scoring tools, such as CNO MRI Scoring tool variants^5^, ^6^ and the Radiologic Index for Nonbacterial Osteitis MRI score,^7^, ^8^ could aid in estimating DA, as WB‐MRI (turbo inversion recovery magnitude or STIR studies) is currently considered the primary imaging method for CNO both at initial diagnosis and during follow‐up.^9^, ^10^ In contrast to the CNO CDAS (CARRA), the PedCNO response score focuses on the number of active lesions assessed by MRI. Several international cohorts have reported that there are usually fewer perceived lesions than MRI‐defined lesions at onset, especially during the course of the disease.^1^, ^11^, ^12^, ^13^, ^14^

**Figure 1:**
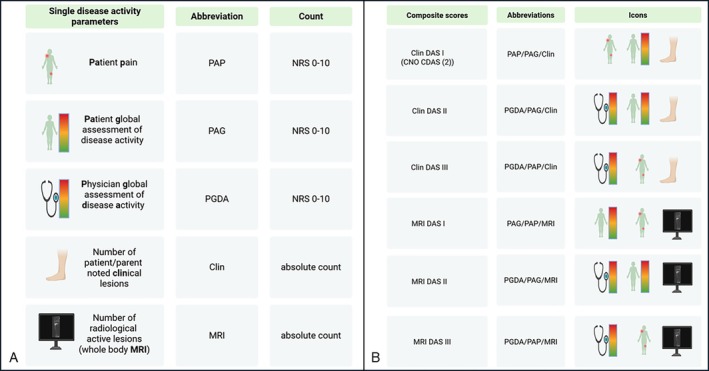
(A) Single disease activity parameters. (B) Composite scores. CDAS, composite disease activity score; Clin, number of clinical lesions; CNO, chronic nonbacterial osteomyelitis; DAS, disease activity score; MRI, number of radiologic lesions by magnetic resonance imaging; NRS, Numeric Rating Scale; PAG, patient global assessment of disease activity; PAP, patient assessment of pain; PGDA, physician global assessment of disease activity.

These currently discussed clinical and radiologic CNO features, as well as single DA components (PGDA, patient assessment of pain [PAP], and PAG) (Figure 1A) and the PedCNO response score (with improvements of 30%, 50%, or 70% of variables) at onset and during the first 4 years of follow‐up, were recently described in a cohort of children and adolescents newly diagnosed with CNO and enrolled in the German National Pediatric Rheumatologic database (NPRD).^13^, ^14^ Because numeric scores may have the advantage of comprehensive usage in daily clinical practice, we have extended the cohort analysis by the CNO CDAS and further composite scores, including the MRI‐based number of lesions and the physician's judgment. These composite scores were calculated on the basis of long‐term registry data and compared in terms of their ability to describe CNO DA, including a proposal for “inactive disease.” NPRD cannot follow treatment efficacy in a controlled setting; nevertheless, the current considerations might have implications for the setup of treat‐to‐target protocols/cohorts or even controlled trials in future international efforts.^6^, ^15^, ^16^, ^17^ A reliable consensus definition for inactive disease or even remission seems crucial for daily clinical practice as well as for “treat‐to‐target” strategies and future comparative studies.^18^

The objective of this study is to propose composite scores for the assessment of DA and to compare the performances with existing composite scores—the proposed CNO CDAS and the PedCNO response score—using data from an established German registry (NPRD). In addition, we suggest a 90% improvement category within the PedCNO response score (PedCNO90). In summary, this study aims to propose different approaches to assess DA in patients with CNO.

## PATIENTS AND METHODS

### Registry and questionnaires

The setup of the NPRD and the clinical follow‐up of the patients with CNO included in this registry have been described recently.^13^, ^14^ No information regarding racial or ethnic affiliation was analyzed in the registry. In summary, two questionnaires were used. The patient questionnaire includes the German version of the C‐HAQ and PAG (as a measure of patient‐reported DA) and PAP, each on a 21‐point NRS ranging from 0 to 10. The physician's questionnaire includes a variety of clinical and sociodemographic features (eg, PGDA, clinical number of bone lesions, number of lesions defined by MRI, and laboratory parameters such as ESR) as well as treatment modalities and MRI imaging analysis. Patients with a disease duration of ≤12 months until first documentation (incident cases) in the registry and at least one follow‐up visit of up to 4 years were considered for inclusion in the long‐term analysis (Table S1).^13^ Patients were selected by expert‐confirmed diagnosis of nonbacterial osteomyelitis with the exclusion of a bacterial, syndromic, or oncologic differential diagnosis, including metabolic bone diseases. The following clinical variables were defined in the long‐term analysis as progression parameters.^14^ Single parameters describing “inactive” disease were proposed, primarily considering DA estimation by the treating physicians with an NRS score <1 (range 0–10), patient estimation of DA or pain (NRS score <1) or a number of active lesions of zero (MRI or clinically).^13^, ^14^

The objective of the current analysis was to propose composite DA measures on the basis of these clinical and imaging data (Figure 1B). The composite assessment tool PedCNO response score has been used to calculate treatment response over time.^1^ Among the five variables, score categories of 30%, 50%, and 70% improvement for this cohort have been reported previously.^14^ A new 90% category of improvement was introduced.

### Statistical analysis

Descriptive statistics, such as absolute and relative frequencies or means and SDs, were used to describe the distributions of sociodemographic, clinical, and imaging parameters (Table S1). Preanalyses of the data were performed to investigate possible attrition bias owing to loss to follow‐up. We could not detect a statistically significant association between collected parameters at baseline and the likelihood of loss to follow‐up.^14^ DA scores and courses were calculated by using linear mixed models. The composite response PedCNO response score was calculated from baseline to each follow‐up assessment (Table 1). Longitudinal data were analyzed via generalized linear mixed models including time and variables of interest as covariates. PedCNO results were calculated by using chi‐square tests, with *P* < 0.05 indicating significance. Inactivity measures (Table 2) were calculated by using chi‐square tests. Statistical analyses were performed with SAS 9.3.

**Table 1:** PedCNO response scores*

	1 YFU			2 YFU			3 YFU			4 YFU		
	N_total_	n	%	N_total_	n	%	N_total_	n	%	N_total_	n	%
PedCNO30	311	155	50	248	154	62	144	79	55	84	59	70
PedCNO50	311	150	48	248	143	58	144	73	51	84	56	67
PedCNO70	311	132	42	248	116	47	144	62	43	84	45	54
PedCNO90	311	120	39	248	105	42	144	59	41	84	41	49

* PedCNO30/50/70/90, Pediatric Chronic Nonbacterial Osteomyelitis 30%/50%/70%/90% response; YFU, years of follow‐up.

**Table 2:** Criteria for inactive disease in all patients and follow‐up*

	Baseline (mean 5.8 months after diagnosis)			1 YFU			*P* value baseline vs 1 YFU	3 YFU			*P* value 1 YFU vs 3 YFU	Prediction of remission (ROC)	Score
	Total N	n	%	Total N	n	%		Total N	n	%			
PGDA <1	279	94	33	279	145	52	<0.001	142	81	57	0.191	–	–
PAG <1	261	64	25	261	105	40	<0.001	126	42	33	0.345	–	–
PAP <1	262	87	33	262	125	48	<0.001	125	63	50	0.501	–	–
Clin = 0	242	86	35	242	120	49	<0.001	137	85	67	0.014	–	–
MRI = 0	240	71	30	240	79	33	0.092	126	63	50	0.008	–	–
PAG/PAP <1 Clin = 0	197	44	20	197	34	17	<0.001	110	29	26	0.078	0.94	CNO CDAS
PAG/PAP <1 MRI = 0	213	41	19	213	35	16	0.084	109	28	26	0.035	0.98	MRI DAS I
PGDA <1 PAG <1 MRI = 0	209	22	10	209	19	9	0.071	109	13	12	0.649	0.98	MRI DAS II
PGDA <1 PAP <1 MRI = 0	206	26	13	206	23	11	0.093	108	13	12	0.301	0.99	MRI DAS III
PGDA <1 PAG <1 Clin = 0	210	25	12	210	20	10	0.114	110	13	12	0.387	0.95	Clin DAS II
PGDA <1 PAP <1 Clin = 0	207	28	13	207	22	11	0.079	109	13	12	0.481	0.94	Clin DAS III

* Inactivity measures (baseline vs follow‐up) were calculated using the chi‐square test. The probability of remission over time was predicted using different scores, and compared through an ROC analysis with the area under the curve values indicating the predictive accurancy of each score. CDAS, composite disease activity score; Clin, number of patient‐/parent‐noted clinical lesions; CNO, chronic nonbacterial osteomyelitis; DAS, disease activity score; MRI, number of radiologic lesions by magnetic resonance imaging; PAG, patient global assessment of disease activity; PAP, patient assessment of pain; PGDA, physician global assessment of disease activity; ROC, receiver operating characteristic; YFU, years of follow‐up.

The study was approved by the ethics committee of the Charité Medical University of Berlin. All participants provided written informed consent/assent. The study protocol is in compliance with the ethical guidelines of the 1975 Declaration of Helsinki. The datasets used and/or analyzed during the current study are available from the corresponding author upon reasonable request.

## RESULTS

### Description of the cohort by using individual parameters as DA scores

**Figure 2:**
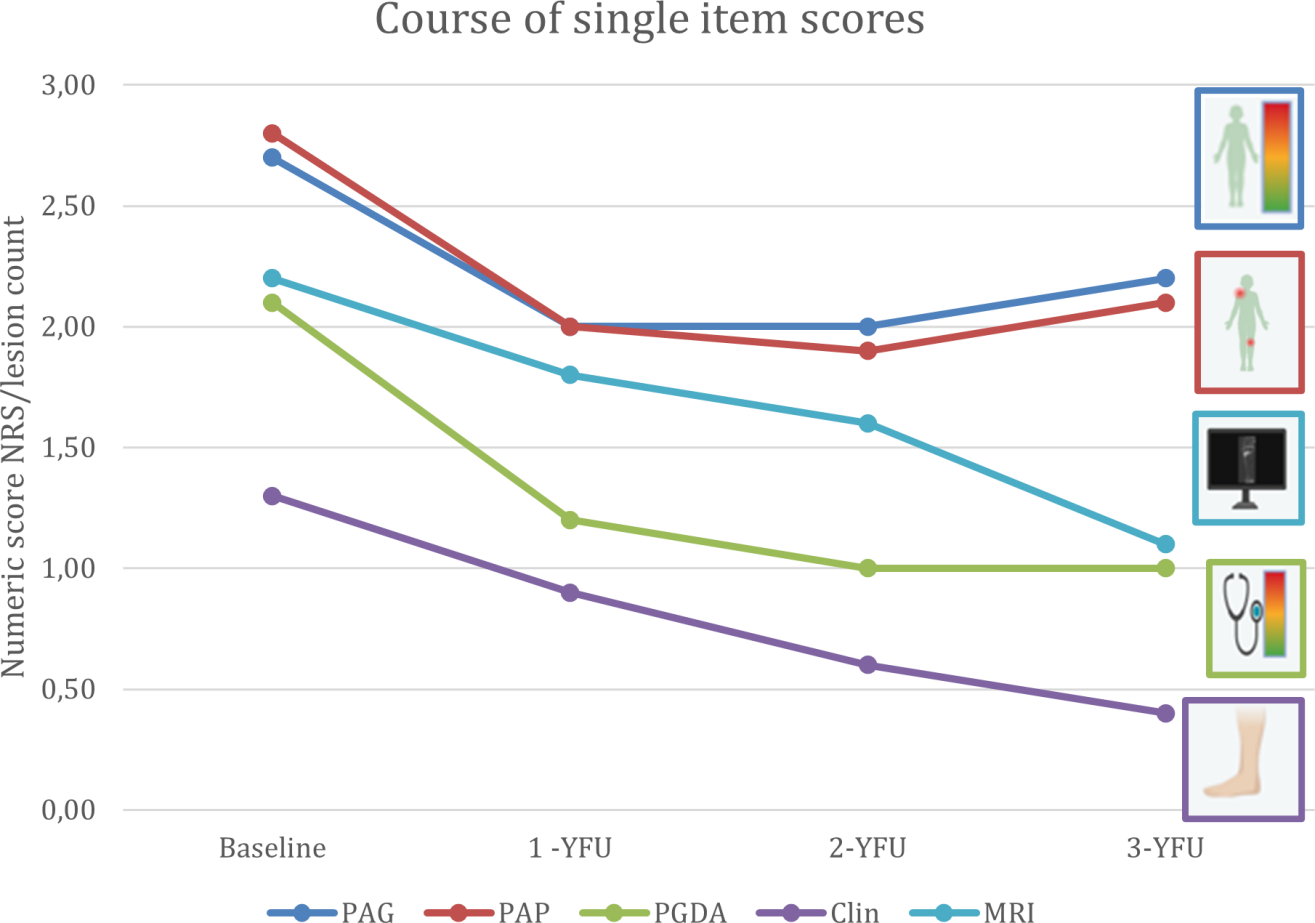
Single‐item disease activity scores during the disease course. Clin, number of clinical lesions; MRI, number of radiologic lesions by magnetic resonance imaging; NRS, Numeric Rating Scale; PAG, patient global assessment of disease activity; PAP, patient assessment of pain; PGDA, physician global assessment of disease activity; YFU, years of follow‐up.

Four hundred patients were enrolled in the NPRD at disease onset, and 145 patients still had a documented 3‐year follow‐up visit. Sixty‐five percent were female, with a mean age of 11.1 years (Table S1). Patients are treated first‐line with nonsteroidal anti‐inflammatory drugs (NSAIDs) with the possibility of extending therapy via common therapeutic agents (eg, according to proposed consensus treatment plans^16^), such as glucocorticoids, bisphosphonates, conventional disease‐modifying antirheumatic drugs (DMARDs; including methotrexate and sulfasalazine), and biologic DMARDs (most commonly etanercept and adalimumab).^13^, ^14^ The results of single DA parameters over time are listed in Figure 2 and Table S2.^14^

Patients in this cohort showed a significant improvement in DA over 1 year of follow‐up when individual parameters were assessed: number of clinical lesions (Clin), MRI‐defined lesions, PGDA, PAP, and PAG (Table S2; Figure 2).^13^, ^14^ However, sustained significant change during follow‐up was observed only in the clinical and MRI lesion counts (Table S2).

### Description of the cohort by using CDAS

**Figure 3:**
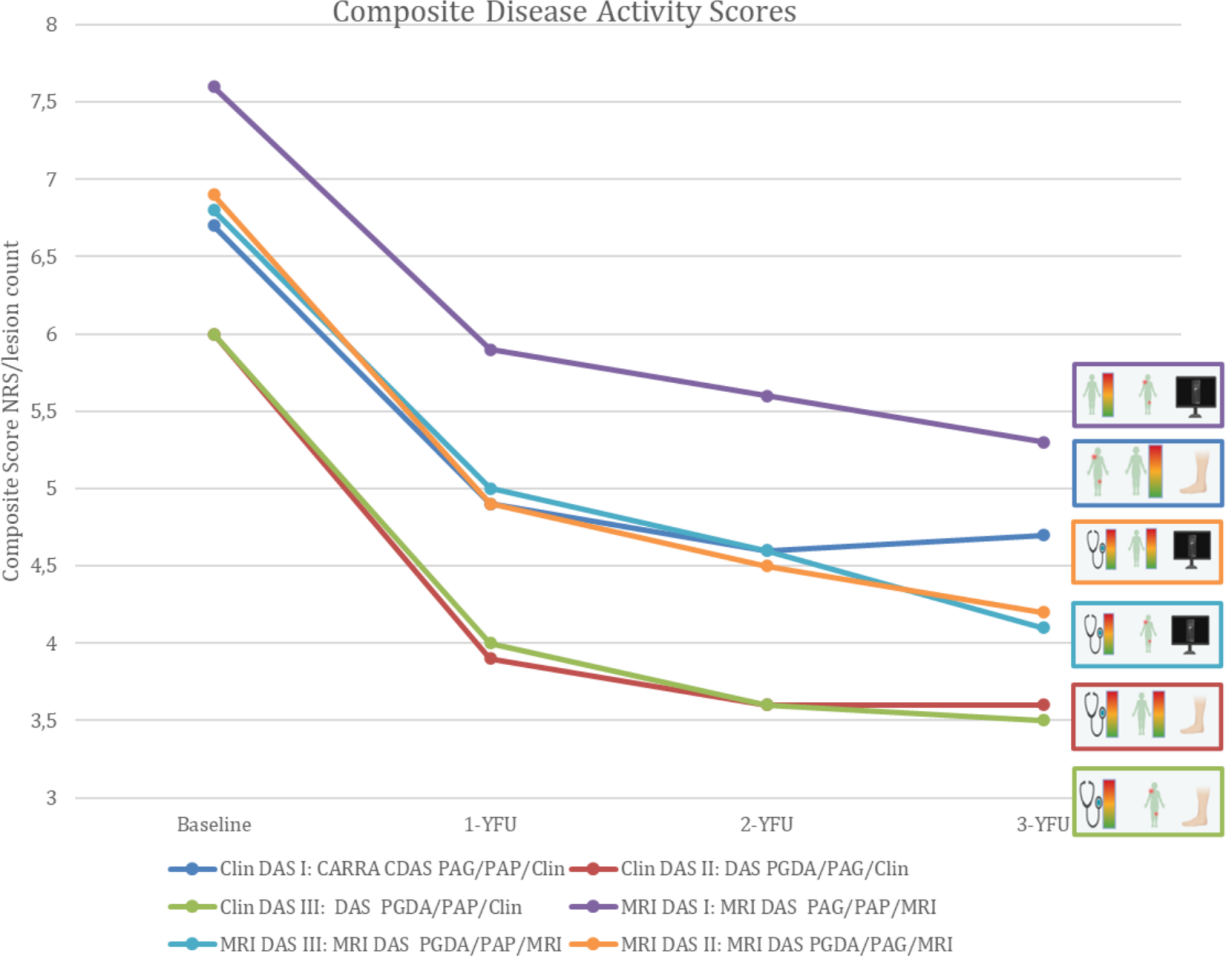
Numeric composite scores during disease course. CARRA, Childhood Arthritis and Rheumatology Research Alliance; CDAS, composite disease activity score; Clin, number of clinical lesions; DAS, disease activity score; MRI, number of radiologic lesions by magnetic resonance imaging; NRS, Numeric Rating Scale; PAG, patient global assessment of disease activity; PAP, patient assessment of pain; YFU, years of follow‐up.

Applying the CNO CDAS (PAG, PAP, Clin), a decrease was noted from baseline/inclusion into the registry to the first follow‐up (*P* = 0.014), whereas no further significant changes were noted during follow‐up afterwards (*P* = 0.899; Figure 3). Notably, the baseline status already reflects 6 months of NSAID therapy.^13^ With respect to the CNO CDAS score, particularly with respect to the parameters of PAG and PAP, no further significant changes were observed after the first year (Figure 3; Table S2).

Applying a variation of the CNO CDAS by exchanging clinical lesions by MRI‐defined lesions, the MRI DAS (PAG, PAP, and MRI lesions) was able to document changes after 1 year. As expected, the overall score levels are higher on the MRI DAS because clinically silent lesions are detected by MRI (Figure 3).

For further refinement using the PGDA instead of the PAG, the Clin DAS II (PGDA, PAP, and Clin) showed the lowest overall scores and lacked responsiveness after 2 years. No relevant changes were detected by exchanging PAP and PAG; thus, both the Clin DAS II/III (PGDA, PAG/PAP, and Clin) scores were similar, because the absolute numbers of PAGs and PAPs over time were almost equal in this cohort. Finally, by including the PGDA and the MRI lesions in addition to the PAG/PAP, the two MRI DAS II/III (PGDA, PAG/PAP, and MRI) scores were calculated. Both scores decreased over the entire follow‐up period. Because the absolute NRS scores of the PAG and PAP were higher than those of the PGDA, those composite scores that include PGDA were lower than the MRI DAS I scores (PAG, PAP, and MRI) (Figure 3). Regarding responsiveness over time (compared with the course of single and other composite scores), the MRI DAS II/III (PGDA, PAG/PAP, and MRI) continuously decreased over time, whereas the CNO CDAS even increased after year 2. Because no gold standard reference benchmark is available for CNO DA measures so far, no comparative testing and validation is currently possible for the MRI DAS II/III (PGDA, PAG/PAP, and MRI).

### Proposed definition of inactive disease

#### Numeric scores

Most single‐item scores with a strict criterion for inactive disease (<1 or 0) changed significantly from baseline to the 1‐year follow‐up. After 3 years, 50% of patients had no MRI lesions, whereas 67% had no clinical lesions; both variables improved significantly over the years. Additionally, 36% of patients reported no complaints or pain. However, most composite scores with the same criterion (<1 or 0) did not reach significance either in the first year or over the entire follow‐up period. Only 12% of patients were considered to have inactive disease (as defined by the combination of clinical or MRI lesions = 0, unremarkable physician assessments, and patient‐reported pain or DA), largely because of elevated subjective score parameters (patient pain/DA) (Table 2).

The probability of remission over time was predicted using different scores and compared through a receiver operating characteristic analysis with the area under the curve values indicating the predictive accurancy of each score. The area under the curve was comparably high in all scores, whereas MRI‐containing scores had a higher predictive accuracy, especially if they also contained PGDA (Table 2).

#### PedCNO score

The PedCNO response score consists of the described five variables; of these variables, score categories of 30%, 50%, and 70% improvement of variables were calculated and already published.^1^, ^14^ We were able to calculate an improvement rate of 90% for the variables. Over the years, constant improvement was noted: 49% of patients experienced a 90% improvement in variables after 4 years (Table 1) (data analysis supported a calculation after 4 years).

## DISCUSSION

In the current analysis, we focused on measurement tools to define CNO DA, including a proposal for minimal or inactive disease using these composites. We analyzed 400 patients with up to four follow‐up visits in specialized centers for pediatric rheumatology. Because no long‐term prospectively controlled trials in CNO exist, there is no clear answer to the urgent questions concerning the superiority of clinical (PGDA, PAG, PAP, and Clin) over MRI‐based estimation of DA. Thus, it is still an open debate whether the patient‐noticed number of lesions or the number of active MRI lesions, including clinically silent lesions as defined by MRI, is the gold standard for therapeutic decisions and the prediction of outcome in the care of patients with CNO. Such a prioritization will be important for the subsequent definitions of inactive disease and remission in CNO.

**Figure 4:**
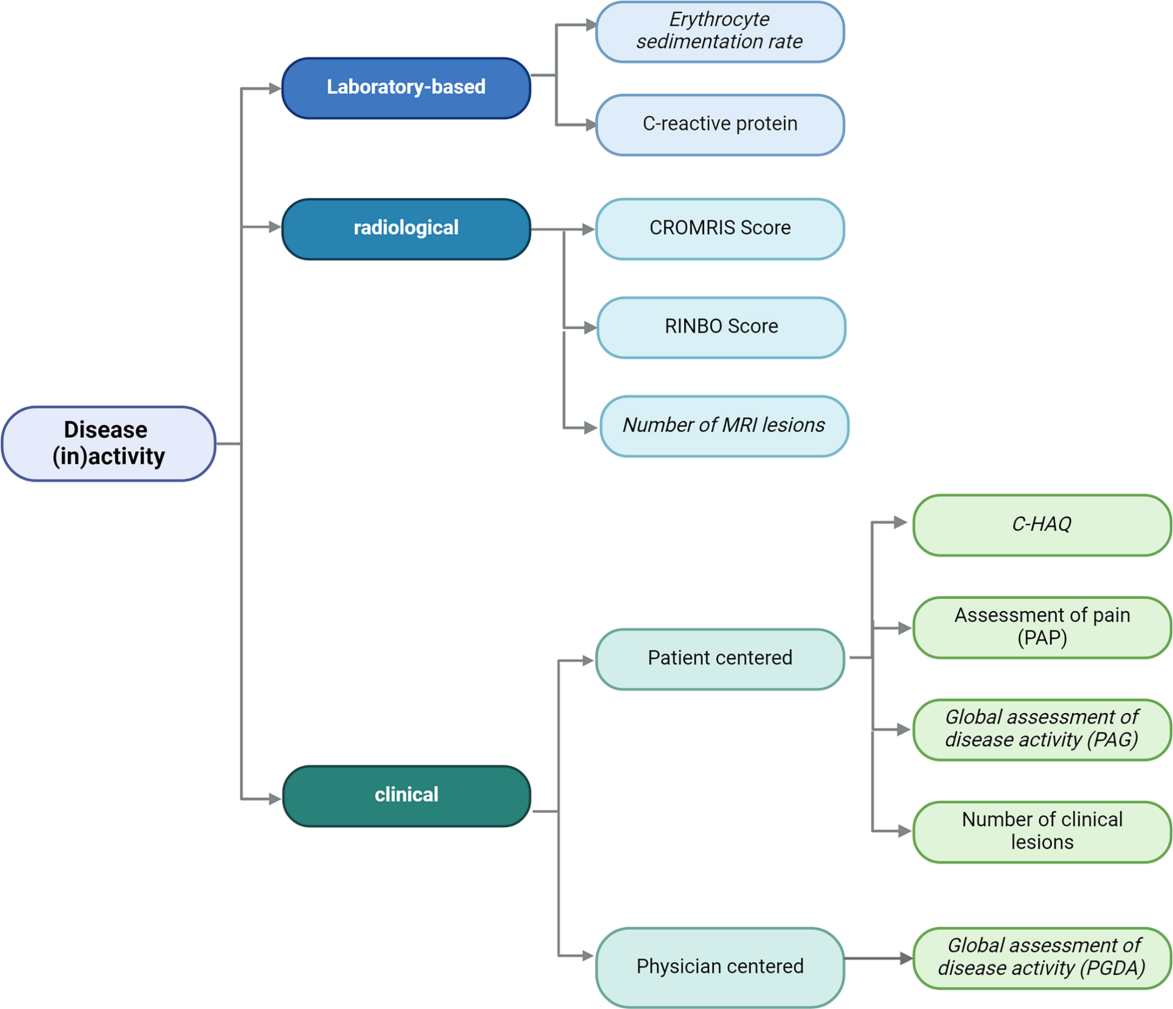
Assessment of disease activity in patients with chronic nonbacterial osteomyelitis. Parameters in italics display the Pediatric Chronic Nonbacterial Osteomyelitis score. C‐HAQ, Childhood Health Assessment Questionnaire; CROMRIS, Chronic Nonbacterial Osteomyelitis Magnetic Resonance Imaging Scoring tool^6^; MRI, magnetic resonance imaging; PAG, patient global assessment of disease activity; PAP, patient assessment of pain; PGDA, physician global assessment of disease activity; RINBO, Radiologic Index for Nonbacterial Osteitis.^8^

The assessment of DA/remission is based on the main columns of clinical and radiologic^5^, ^6^, ^8^, ^19^ evaluation in combination with the assessment of systemic inflammation. Although specific laboratory parameters are lacking, standard inflammation parameters such as C‐reactive protein and ESR are routinely used (Figure 4).^20^, ^21^ The use of individual activity parameters alone, however, may fall short in adequately capturing the complex nature of the disease in many scenarios.^14^ Consequently, composite scores have been developed. Such scores are widely used in clinical settings to provide a comprehensive overview of a patient's health status by synthesizing multiple individual parameters into a unified metric. This methodology is particularly effective and may better capture the complexity of diseases such as CNO through the use of CDASs.^22^

In the initial diagnostic setup of a patient with skeletal pain, the absence of MRI lesions effectively excludes CNO disease, as WB‐MRI is considered the gold standard for diagnosing and monitoring CNO.^10^, ^15^, ^23^, ^24^, ^25^ In such cases, alternative causes for the patient's symptoms should be explored. During the course of CNO disease, the interpretation of clinically silent MRI lesions can be thrilling, as these lesions may remain unnoticed by patients and parents but could subsequently develop into clinically significant painful lesions and structural deficits. Conversely, patients often report rapid clinical improvement within a few weeks or months of treatment, resulting in ambiguous assessments of treatment responsiveness. Thus, changes in MRI may lag behind the patient's perception and/or physician‐considered improvement of disease. However, MRI changes may, in contrast, remain visible over years of follow‐up.^7^, ^11^, ^14^, ^26^ In a previous long‐term observational study of a single‐center German CNO cohort, half of the 93 patients no longer noticed their lesions after 6 months of treatment, whereas only 4% had no detectable lesions on MRI at the same time.^11^ Clinical experience has shown that “early” cessation of therapy at times when patients experience significant improvement after 3 to 6 months often results in subsequent clinical flare‐ups or a chronic disease course.^26^, ^27^, ^28^

From a clinical perspective, CDASs that include MRI lesion counts and physicians’ assessment appear to better define DA compared with single parameters, which may underestimate or overestimate it. Composite scores need to (1) include objective parameters and (2) avoid one‐sided overweighting of clinical, radiologic, or laboratory parameters to improve objectivity. Although patient‐reported outcomes like PAG or PAP capture subjective assessments, incorporating active MRI lesion count introduces a more objective component. MRI‐inclusive scores demonstrated a more pronounced decline over time, as illustrated in Figure 3. However, persistent PAP scores in the NPRD registry suggest overestimation of DA in patients with pain amplification.

The CNO CDAS incorporates active clinical lesion count as a third component alongside PAG and PAP. Its validation, based on CHronic nonbacterial Osteomyelitis International Registry (CHOIR) data, included patient surveys on difficulties using body parts, fatigue, mood issues, and self‐assessed DA.^2^ The authors concluded that the CNO CDAS is a reasonable tool to describe changes in DA and therapeutic responsiveness in patients with CNO,^2^ although objective parameters are still lacking.

In this study, we proposed composite scores analogous to the CNO CDAS with three components, including MRI lesion counts, to improve objectivity and facilitate comparability in clinical trials. Over the follow‐up period, MRI‐inclusive scores consistently decreased, whereas patient‐reported lesion‐based scores were less responsive after 2 years and even worsened after 3 years, likely because of fast improvement after administration of anti‐inflammatory drugs. This may reduce the significance of these clinical parameters in long‐term follow‐up.

In addition, in the presented cohort, approximately half of the patients indicated pain (NRS >1) after 1 year, and the mean pain score was consistently greater (NRS mean values of approximately 2). PAG and PAP measurements revealed quite comparable data. In particular, pain assessment limits the use of the PAG and PAP measures, especially when they are combined into one composite score. Among patients with unremarkable WB‐MRI at the 3‐year follow‐up, only 25% reported a PAG score <1, and 45% reported a PAP score <1 (49% and 56% when using an NRS <2). This suggests that long‐term CNO cohorts may have a tendency toward pain amplification. Therefore, we propose that, with regard to responsiveness, the MRI DAS II/III (PGDA, PAG or PAP, and MRI) seems to best reflect the CNO DA over time in the current cohort. The attempt to define a strict composite score for inactive disease using a cutoff of NRS <1 resulted in a limited number of patients reaching inactive disease (Table 2; Table S3), even when only MRI‐negative patients were considered for calculation (Table S3). To define remission as accurately as possible despite these limitations, we considered remission as the absence of MRI lesions, absence of clinical lesions, a PGDA score <1, and a PAG score <1. Defining remission solely by clinical lesions or solely by MRI findings could introduce bias in favor of the respective assessment method. However, the attempt to describe inactive disease by composite scores in this cohort falls short because patients’ consideration of DAs and pain seems limited owing to the unexpectedly high prevalence of pain.

The long‐term clinical relevance of either the number of lesions defined by MRI or patient‐identified lesions is currently open for discussion. Such clarification will be crucial for defining inactive disease and remission in patients with CNO.

The PedCNO response score seems to be a reasonable relative tool for estimating relative DA and treatment response over time for the individual patient in this registry. However, the PedCNO score result does not estimate the initial severity.^1^, ^14^ As shown in Table 1, the percentages of patients reaching a PedCNO90 increase over time, which is consistent with the decreasing number of patients with active disease (defined by MRI‐counted lesions; clinical lesions; PGDA <1, and PAG/PAP <1) over time (Table 2). More than 39% of patients reach a PedCNO90 during the first year, increasing to 49% after 4 years. Allowing one component to worsen may be reasonable in patients exhibiting pain amplification, as demonstrated in the current analysis. To limit such a bias, the PedCNO90 score was introduced as an even more stringent parameter to the existing PedCNO70 to enforce disease inactivity. Therefore, pedCNO90 might provide a valuable estimation of minimal DA, particularly for patients with minimal or no MRI‐detected lesions. Of note, PedCNO is not designed and validated for decision‐making in daily clinical practice; however, it helps to describe the multifacetted changes in DA over time.

The absence of a treat‐to‐target design in the registry limits our ability to conclude whether more aggressive treatment strategies might have led to further improvement in the registered patients. The generalizability of the findings is limited owing to several factors in this registry: selection bias of centers and visits (documentation only once per year), the uncontrolled setting of the registry with possible bias of documentation, lack of validated treatment recommendations, and missing data on socioeconomic or ethical issues.

The proposed scores aim to stimulate discussion on various aspects of assessing DA, providing a basis for future considerations. Refinement of these scores is necessary to develop reliable measurement tools that allow a more precise grading of the disease. This, in turn, will facilitate the design of clinical trials with well‐defined and reliable endpoints.

Further studies are needed to verify these results in a prospective cohort or, ideally, in controlled trials. CDASs in combination with “risk” parameters for a severe disease course, such as an elevated ESR, a multifocal course, or particular bone site inflammation at baseline,^14^ might help physicians to make treatment decisions. Comparative trials are needed to identify valid modes to describe DA and inactive disease.^18^ Here, we propose that the use of MRI‐based scores, together with physicians’ assessment of DA, is relevant for the estimation of DA.

The primary limitation of this study is the absence of an external benchmark for DA, which could have served as the gold standard for validating the proposed scoring system. The analysis is based on a registry with annual visits, which may reduce data precision. Delays in initial data entry likely introduced bias, especially because therapeutic responses estimated by PAG and PAP in the first months can be substantial. Moreover, a significant number of patients dropped out of the registry, limiting the ability to observe them for the full 4‐year period and potentially introducing a bias. Pain amplification in CNO may diminish the reliability of patient‐reported outcomes in the long run, both in individual assessments and composite score analyses. Treatment based on patient‐centered scores alone may lead to early cessation of treatment, because these scores tend to decrease sooner and faster than MRI‐based scores. In contrast, if pain amplicifation is present, unnecessary treatment prolongation may result. Future trials should address this bias as well as a potential overtreatment when focusing on silent MRI lesions.

For the strict definition of disease inactivity and remission we have not included stakeholders’ assessment for the chosen cut‐offs so far. Overall patient/parent opinion is reflected in the C‐HAQ and in the PAG and PAP as described by Reiser et al.^14^

Although the registry collects data prospectively, the registry can only document treatment in an uncontrolled setting. Although this is a limitation for data interpretation, the results show that there is a need to conduct prospective controlled trials. The regional homogeneity of the cohort may limit the generalizability of our findings to more diverse populations. Additionally, no data on race and ethnicity were collected in this registry, which restricts our ability to comprehensively evaluate the impact of demographic diversity on DA and outcomes.

Different composite scores for assessment of DA in PedCNO are proposed. Composite scores consisting of a combination of clinical (patient and physician‐based) and MRI‐based judgments are more objective than clinical composite scores. Future trials are necessary to validate these results.

## AUTHOR CONTRIBUTIONS

All authors contributed to at least one of the following manuscript preparation roles: conceptualization AND/OR methodology, software, investigation, formal analysis, data curation, visualization, and validation AND drafting or reviewing/editing the final draft. As corresponding author, Dr Reiser confirms that all authors have provided the final approval of the version to be published and takes responsibility for the affirmations regarding article submission (eg, not under consideration by another journal), the integrity of the data presented, and the statements regarding compliance with institutional review board/Declaration of Helsinki requirements.

## ROLE OF THE STUDY SPONSOR

The funders had no role in the study design, data collection, data analysis, interpretation of the data, writing of the report, or decision to publish.

## Supporting information


**Data S1:** Supplementary Information

**Disclosure form**.
