# Supplementary material for: Assessing Disease Activity in Pediatric Chronic Nonbacterial Osteomyelitis: A Proposal for Composite Scoring, Including Inactivity Measures
Source: ACR Open Rheumatol. 2025 Jul 2;7(7):e70061. doi: 10.1002/acr2.70061 (PMC12221992; doi:10.1002/acr2.70061)
Supplement: Supplementary file 1 — Data S1: Supplementary Information [file ACR2-7-e70061-s001.docx]

**Supplement**

Supplement Table 1: Sociodemografic and clinical features of patients at inclusion into the registry

|  | | | |  |
| --- | --- | --- | --- | --- |
| Total |  | N=400 | | |
|  |  | N _related_ | n | % |
| Sex |  | 400 |  |  |
|  | female |  | 258 | 64,5 |
|  | male |  | 142 | 35,5 |
| Age, in years; mean (STD), median |  | 400 | 11.1 (3.0), 11 | |
| disease duration in months till inclusion; mean (STD), median |  | 400 | 10.9 (6.4), 10 | |
| disease duration in months till first rheumatology assessment; Mean (STD), median |  | 280 | 5.3 (5.2), 3 | |
| HLA-B27 |  | 344 |  |  |
|  | negative |  | 138 | 40,1 |
|  | positive |  | 21 | 6,1 |
|  | not done |  | 185 | 53,8 |
|  |  |  |  |  |
| ESR in mm/h; Mean (STD), median |  | 251 | 16.2 (15.0), 11 | |
| C-HAQ; mean (STD), median |  | 361 | 0.3 (0.4), 0.125 | |
|  |  |  |  |  |
| cumulative therapy till inclusion |  | 400 |  |  |
|  | any |  | 387 | 96,8 |
|  | none |  | 13 | 3,3 |
|  | NSAIDs |  | 344 | 86,0 |
|  | Glucocorticoids, < 0,2 mg per kg body weight |  | 21 | 5,3 |
|  | Glucocorticoids, ≥ 0,2 mg per kg body weight |  | 33 | 8,3 |
|  | Bisphosphonates |  | 34 | 8,5 |
|  |  |  |  |  |
| DMARD therapy till inclusion |  | 341 |  |  |
|  | any |  | 56 | 16.4 |
|  | csDMARD |  | 43 | 12,6 |
|  | MTX |  | 30 | 8,8 |
|  | Sulfasalazine |  | 13 | 3,8 |
|  | bDMARD |  | 21 | 6,1 |
|  | Etanercept |  | 10 | 2,9 |
|  | Adalimumab |  | 11 | 3,2 |

Supplement Table 2: Progression of single DA parameters.

|  | Baseline  N=400 | 1-YFU  N=313 | P value Baseline vs 1-YFU | 2-YFU  N=248 | 3-YFU  N=145 | P value 1-YFU vs 3-YFU |
| --- | --- | --- | --- | --- | --- | --- |
| PAG | 2.7 (2.5) | 2.0 (2.1) | <0.001 | 2.0 (2.2) | 2.2 (2.3) | 0.628 |
| PAP | 2.8 (2.8) | 2.0 (2.5) | <0.001 | 1.9 (2.5) | 2.1 (2.6) | 0.815 |
| PGDA | 2.1 (1.9) | 1.2 (1.5) | <0.001 | 1.0 (1.5) | 1.0 (1.4) | 0.242 |
| Clinical lesions | 1.3 (1.9) | 0.9 (1.2) | 0.020 | 0.6 (1.1) | 0.4 (0.7) | **0.003** |
| MRI lesions | 2.2 (2.8) | 1.8 (2.5) | 0.005 | 1.6 (2.5) | 1.1 (1.5) | **0.004** |

YFU: year of follow-up; PAG: patient global assessment of disease activity; PAP: patient assessment of pain. PGDA: physician global assessment of disease activity; Significant changes over time are indicated by p< 0.05. Comparisons of Baseline versus 1-YFU and 1-YFU versus 3-YFU were estimated using estimated means from generalized linear mixed models

Supplement Table 3: Inactive or minimal disease in patients with **zero MRI lesions**

|  |  | | | | | | | |
| --- | --- | --- | --- | --- | --- | --- | --- | --- |
|  | Baseline | | 1-YFU | | 2-YFU | | 3-YFU | |
|  | n | % | n | % | n | % | n | % |
|  |  |  |  |  |  |  |  |  |
| PAG/PAP < 1 / Clin=0 | 13 | 21 | 24 | 29 | 28 | 31 | 10 | 18 |
| PAG/PAP < 1 / MRI=0 | 14 | 22 | 25 | 30 | 31 | 35 | 12 | 22 |
|  |  |  |  |  |  |  |  |  |
| PGDA<1 PAG<1 MRI=0 | 17 | 27 | 38 | 45 | 38 | 43 | 20 | 36 |
| PGDA<1 PAP<1 MRI=0 | 19 | 30 | 40 | 48 | 43 | 48 | 23 | 42 |
|  |  |  |  |  |  |  |  |  |
| PGDA<1 PAG<1 Clin=0 | 13 | 21 | 24 | 29 | 30 | 34 | 10 | 18 |
| PGDA<1 PAP<1 Clin=0 | 14 | 22 | 25 | 30 | 33 | 37 | 12 | 22 |
